# Ultrasonographic findings relating to lymph node metastasis in single micropapillary thyroid cancer

**DOI:** 10.1186/1477-7819-12-273

**Published:** 2014-08-28

**Authors:** Yoon Se Lee, Yun-Sung Lim, Jin-Choon Lee, Soo-Geun Wang, Seok-Man Son, Sang-Soo Kim, In-Ju Kim, Byung-Joo Lee

**Affiliations:** Department of Otolaryngology, Asan Medical Center, University of Ulsan College of Medicine, 88 Olympic-ro 43-gil, Songpa-gu, Seoul 138-739 Republic of Korea; Department of Otorhinolaryngology-Head and Neck Surgery, Pusan National University School of Medicine and Medical Research Institute, Pusan National University Hospital, 1-10, Ami-dong, Seo-gu, Pusan, 602-739 Republic of Korea; Department of Internal Medicine, Pusan National University School of Medicine and Medical Research Institute, Ami-dong 1ga, Seo-gu, Pusan 602-739 Republic of Korea

**Keywords:** Papillary thyroid cancer, Ultrasonography, Cervical lymph nodes, Metastasis

## Abstract

**Background:**

In thyroid cancer, preoperative ultrasonography (US) is performed to detect the primary tumor and lymph node metastasis (LNM), which are related to prognosis. This study examined the relationships between specific US findings and LNM in micropapillary thyroid cancer (MPTC).

**Methods:**

Data on 220 patients with solitary MPTC who underwent total thyroidectomy and neck dissection between 2008 and 2009 were evaluated retrospectively. We classified the US findings according to the nature, shape, echogenicity, extent, margin, and calcification of the primary tumor and evaluated the correlations between these findings and those of LNM.

**Results:**

Hypoechogenicity (odds ratio = 2.331, *P* = 0.025) and marked hypoechogenicity (OR = 4.032, *P* = 0.016) of MPTC were risk factors for central LNM. All of the patients with lateral cervical LNM showed hypoechogenicity or marked hypoechogenicity. Hypoechogenicity (odds ratio = 5.349, *P* = 0.047) and other types of calcification (odds ratio = 2.495, *P* = 0.010) were significant risk factors for lateral cervical LNM.

**Conclusions:**

Specific sonographic findings (hypoechogenicity or marked hypoechogenicity, and calcification) suggest LNM.

## Background

Various clinical factors are related to lymph node metastasis (LNM) of papillary thyroid cancer (PTC), including tumor size and extrathyroidal extension. The reported LNM rate of PTC is 20 to 50% [1], and a 90% micrometastasis rate was reported depending upon the detection method [2]. The locoregional recurrence rate of PTC ranges from 5 to 20%, and most recurrences involve lymph nodes [3]. Although micropapillary thyroid cancer (MPTC) usually has a much better prognosis than thyroid cancer of more than 1 cm, LNM is relatively common [4]. The central lymph node metastasis (CLNM) rate and lateral cervical lymph node metastasis (LCLNM) rate in MPTC ranges from 13 to 43% and 3 to 44.5%, respectively [5–8]. Some clinicopathological characteristics of MPTC have been reported to predict aggressive tumor behavior, such as LNM, and some of them have been used as guidelines for surgical extent and adjuvant radioactive iodine therapy [9]. Despite the controversy over the prognostic role of CLNM, LCLNM is a significant predictor of recurrence and distant metastasis [10, 11]. However, the prognostic value of LCLNM is still unclear, mainly due to the slow progression of metastatic lymph nodes and difficulty proving a survival benefit after neck dissection. Therapeutic neck dissection is indicated for definite metastasis in the lateral compartment to reduce the recurrence rate, whereas prophylactic lateral cervical lymph node dissection fails to result in recurrence-free survival and is not usually performed [9].

Preoperative diagnosis of LCLNM determines the treatment strategy, and optimum therapy will improve treatment outcome. Tumor size, extrathyroidal extension, and the number of CLNMs are known predictors of LCLNM in MPTC [11–13]. However, most of these histological factors are determined after surgery and a precise preoperative evaluation is required.

Ultrasonography (US) is widely used in preoperative or follow-up examinations to detect primary and metastatic tumors in PTC and provides critical information on surgical extent [14–16]. US investigations may detect local recurrences at an early stage and can precede either an elevated serum stimulated thyroglobulin level or positive findings on whole-body iodine scans [17]. In addition to diagnostic accuracy, US has the benefit of being a non-invasive, relatively simple procedure, which can be combined with fine needle aspiration (FNA). Recent studies have suggested that some sonographic findings of the primary tumor in PTC are negative prognostic indicators, such as extrathyroidal extension or CLNM [18]. Based on these ideas, this study examined US findings of LNM in MPTC by analyzing the correlations between specific US findings of the primary tumor and LCLNM in PTC.

## Methods

### Patients

Between January 2008 and November 2009, 389 patients were diagnosed with PTC and underwent a total thyroidectomy and routine bilateral central lymph node dissection with or without LNM. Medical charts were reviewed after approval by the institutional review board. The clinical variables collected included age, gender, pathological findings, and US findings. Pathological findings included tumor size, extrathyroidal extension, and LNM (CLNM and/or LCLNM). Patients with solitary MPTC and preoperative US reports were included, whereas all patients with recurrent or multiple PTC or tumors larger than 1 cm were excluded. Central lymph nodes and lateral cervical lymph nodes were classified according to the TNM classification established by the International Union Against Cancer and the American Joint Commission on Cancer (2010 AJCC Seventh Edition) [19].

### Imaging

**Figure 1:**
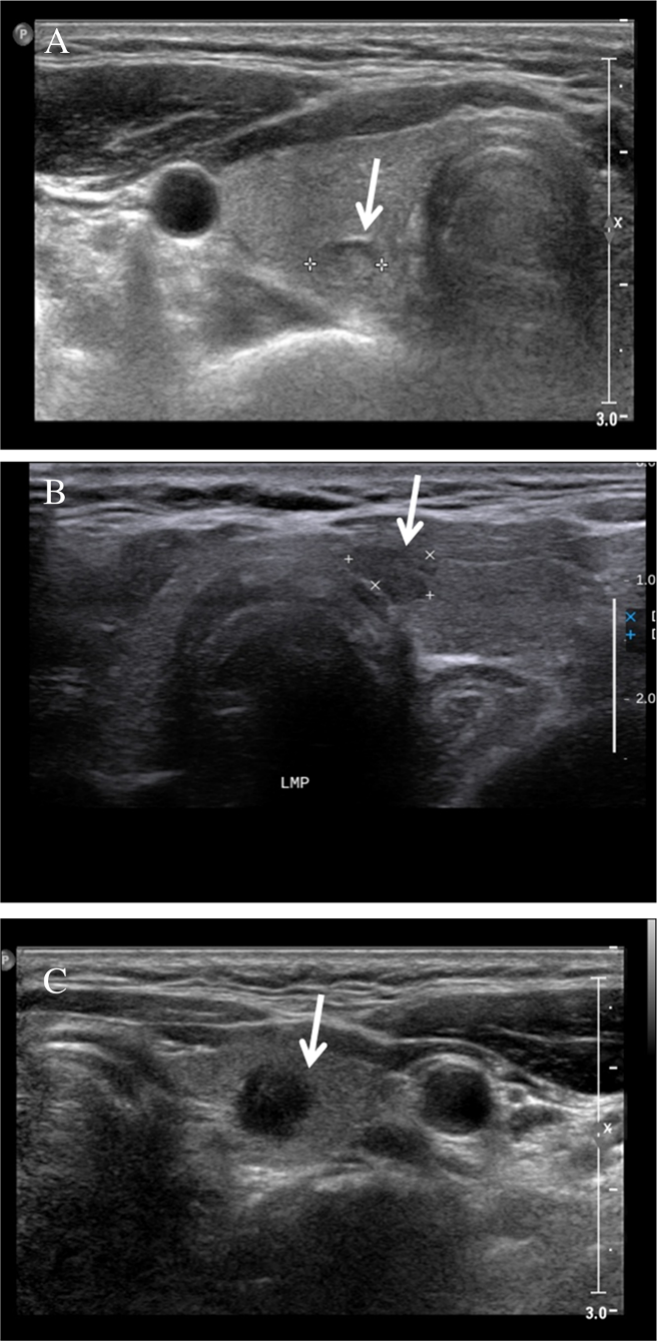
**Echogenicity of thyroid cancer (arrow). (A)** Isoechoic nodule on the posterior aspect of the right thyroid gland. The nodule has similar echogenicity to the thyroid gland. **(B)** Hypoechoic nodule on the medial aspect of the left thyroid gland. The nodule has hypoechoic echogenicity compared to the thyroid gland. **(C)** Markedly hypoechoic nodule of the left thyroid gland. The nodule has more hypoechoic echogenicity than that of strap muscle.

**Figure 2:**
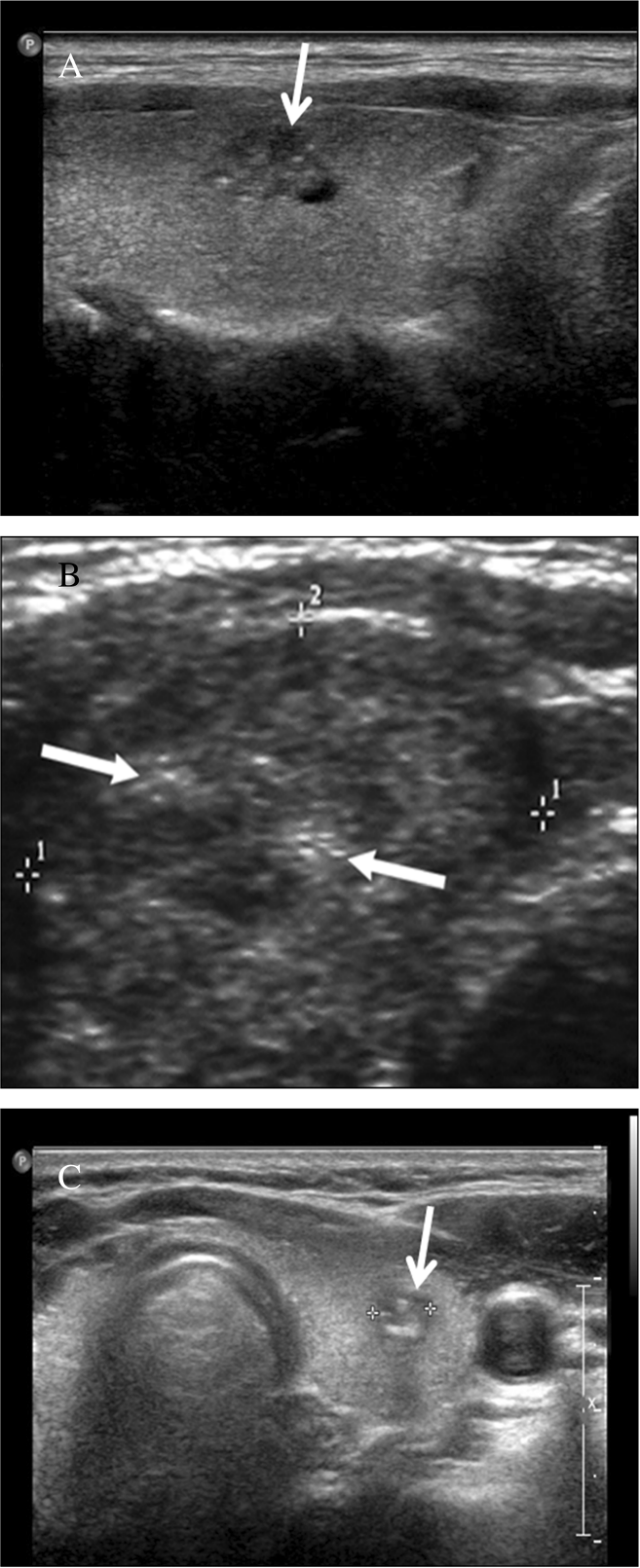
**Calcification of thyroid cancer (arrow). (A)** Microcalcification. **(B)** Other types of calcification. **(C)** Macrocalcification in other types of calcification.

Three experienced endocrinologists performed and reviewed preoperative US examinations of the neck. US findings were documented according to the criteria discriminating malignant from benign thyroid nodules [14]. LNM was confirmed by a pathologist postoperatively. The US examination was performed using a Vivid i US scanner (GE Healthcare, Milwaukee, WI, USA) using electronically focused linear probes with a bandwidth of 10 to 13 MHz [14]. US findings of the tumors were classified according to the nature (solid/mixed), shape (round to ovoid/irregular/taller than wide), echogenicity (isoechoic/hypoechoic/markedly hypoechoic; Figure 1), margin (well defined/poorly defined), calcification (no calcification/microcalcification/other types of calcification; Figure 2), and vascularity of the primary tumor. The sonographic size of the primary tumor was measured. Markedly hypoechoic echogenicity was described when echogenicity of the primary tumor was lower than the that of surrounding strap muscle [14]. Other types of calcification included macrocalcification and fine stippled psammomatous calcification (nodular, piece-like, rim-like, and irregular shape) [20]. These specific US findings were evaluated in terms of the rates of CLNM and LCLNM. Vascularity was excluded in this study because it was not consistent and was not recorded routinely.

### Surgery

Enrolled patients underwent a total thyroidectomy and routine bilateral central lymph node dissection. Central lymph node dissection involved all soft tissues containing lymph nodes, from the hyoid bone superiorly to the innominate artery inferiorly and the common carotid artery laterally [21]. Level II to IV lateral cervical lymph node dissection was performed when US-guided FNA confirmed LNM or when preoperative high-resolution US or neck computed tomography showed suspicious findings. Suspicious findings on US or computed tomography included LCLNM of enlarged size, cystic changes, round shape, eccentric cortical widening, decreased echogenicity, loss of fatty hilum, presence of calcification, and increased intranodal vascularity [22]. A minor axis greater than 50% of the major axis, minor axis greater than 10 mm, or hyperechogenicity with or without microcalcifications were also considered suspicious findings for LNM in PTC [23].

### Statistics

The statistical analyses were performed using SPSS version 15.0 (Chicago, IL, USA). Fisher’s exact test was used to analyze the relationship between the rate of LNM and the US findings. After validating significant US findings, multiple logistic regression analysis was used to identify US findings related to LNM with significant odds ratios. All reported *P*-values are two-sided.

## Results

Of the patients enrolled in this study, 220 of 389 (56.6%) had solitary MPTC and 81.1% of these (*n* = 194) were female. The mean age was 48.4 ± 10.64 years. CLNM and LCLNM were confirmed in 41.4% (*n* = 91) and 6.4% (*n* = 14), respectively. One skip metastasis, defined as LCLNM without CLNM, was observed in this population. The US characteristics are reported in Table 1. Most of the primary tumors were solid (99.5%), hypoechoic (90.9%), with poorly defined margin (73.2%) and absent calcification (74.1%). Irregular, round to ovoid, and taller than wide shape were almost evenly distributed.

**Table 1:** **Patient distribution according to the ultrasonography findings of the primary tumor**

Ultrasonography findings		***n***(%)
Nature	Solid	219 (99.5)
	Mixed	1 (0.5)
Shape	Round to ovoid	83 (37.7)
	Irregular	81 (36.8)
	Taller than wide	56 (25.5)
Echogenicity	Isoechoic	20 (9.1)
	Hypoechoic	80 (36.4)
	Markedly hypoechoic	120 (54.5)
Margin	Well defined	59 (26.8)
	Poorly defined	161 (73.2)
Calcification	Absent	163 (74.1)
	Microcalcification	29 (13.2)
	Other types	28 (12.7)

First, the relationship between the US findings of the primary tumor and the rate of LNM was analyzed (Table 2). In the univariate analyses, solid nature and calcification did not increase the rate of LNM. The primary tumors that were irregular or taller than wide shape showed higher rates of LNM than those that were round to ovoid. A poorly defined margin was significantly related to LNM. We performed multivariate analysis using significant factors from the univariate analysis to reveal the factors predisposing to LNM. Hypoechoic (odds ratio = 2.331, 95% CI 0.073 to 8.839; *P* = 0.025) and markedly hypoechoic (odds ratio = 4.032, 95% CI 0.216 to 16.852; *P* = 0.016) lesions increased the risk of LNM, as compared to isoechoic lesions (Table 3).

**Table 2:** **Univariate analysis of the correlation between ultrasonography findings of the primary tumor and rate of cervical lymph node metastasis in micropapillary thyroid cancer**

Ultrasonography findings		***n***(rate of lymph node metastasis, %)	***P***value
Nature	Solid	91 (41.6)	0.400
	Mixed	0 (0)	
Shape	Round to ovoid	27 (32.5)	0.014
	Irregular	32 (39.5)	
	Taller than wide	32 (57.1)	
Echogenicity	Isoechoic	4 (20.0)	<0.001
	Hypoechoic	23 (28.7)	
	Markedly hypoechoic	64 (53.3)	
Margin	Well defined	18 (30.5)	0.033
	Poorly defined	73 (45.3)	
Calcification	Absent	65 (39.9)	0.745
	Microcalcification	13 (44.8)	
	Other types	13 (46.4)

**Table 3:** **Multivariate analysis presenting correlation between ultrasonography findings of the primary tumor and cervical lymph node metastasis in micropapillary thyroid cancer**

	B	SE	***P***value	Exp(B)	95% CI	
					Lower	Upper
Shape			0.364			
Irregular	-0.465	0.441	0.292	1.592	0.264	1.492
Taller than wide	-0.509	0.371	0.170	1.663	0.290	1.245
Echo			0.014			
Hypoechoic	-1.393	0.621	0.025	2.331	0.073	8.839
Markedly hypoechoic	-0.847	0.350	0.016	4.032	0.216	16.852
Margin	0.095	0.415	0.820	1.099	0.487	2.481
Constant	0.250	0.855	0.770	1.284		

Next, we explored the relationship between the US findings of microPTC and the rate of LCLNM. LCLNM was found in 14 patients (6.4%). Gender and age were not related to LCLNM. Although all of the tumors with LCLNM were solid, the nature and margin were not significant factors affecting LCLNM in the univariate analysis (Table 4). Instead, taller than wide shape seemed to be related to LCLNM (*P* = 0.088), but the rate of LCLNM was not significantly higher than that for either round to ovoid or irregular masses. The primary tumors with LCLNM were all hypoechoic and the rate of LCLNM was higher in “markedly hypoechoic” tumors (10.0%) than just “hypoechoic’ tumors” (2.5%, *P* = 0.049). Of the tumors with LCLNM, 92.9% (*n* = 13) had poorly defined margins, but this was not significantly (*P* = 0.071) related to the rate of LCLNM. Other types of calcified lesions in the primary tumor were related to the rate of LCLNM (*P* = 0.027). After the univariate analysis, multivariate analysis was performed using the relevant factors, shape, echogenicity, margin, and calcification (Table 5). Isoechoic lesions were not seen in tumors with LCLNM and the rate of LCLNM was compared between markedly hypoechoic and hypoechoic lesions. In the multivariate analysis, marked hypoechogenicity (odds ratio = 5.349, 95% CI 1.025 to 27.915; *P* = 0.047) and other calcified lesions (odds ratio = 2.495, 95% CI 1.246 to 4.995; *P* = 0.010) were significant risk factors for LCLNM. The mean size of the tumors with LCLNM (0.71 ± 0.18 cm) was greater than that of the tumors with (0.68 ± 0.18 cm) or without (0.66 ± 0.17 cm) CLNM, but the difference was not significant.

**Table 4:** **Univariate analysis of correlation between ultrasonography findings of the primary tumor and rate of lateral cervical lymph node metastasis in micropapillary thyroid cancer**

Ultrasonography findings		***n***(rate of lateral cervical lymph node metastasis, %)	***P***value
Nature	Solid	14 (6.39)	0.794
	Mixed	0 (0)	
Shape	Round to ovoid	3 (3.61)	0.088
	Irregular	4 (4.94)	
	Taller than wide	7 (12.5)	
Echogenicity	Isoechoic	0 (0)	0.049
	Hypoechoic	2 (2.5)	
	Markedly hypoechoic	12 (10)	
Margin	Well defined	1 (1.69)	0.071
	Poorly defined	13 (8.07)	
Calcification	Absent	8 (4.9)	0.027
	Microcalcification	1 (3.4)	
	Other types	5 (6.4)

**Table 5:** **Multivariate analysis presenting correlation between ultrasonography findings of the primary tumor and lateral cervical lymph node metastasis in micropapillary thyroid cancer**

Ultrasonography findings	B	SE	***P***value	Exp (β)	95% CI	
					Lower	Upper
Shape	0.158	0.448	0.724	1.172	0.487	2.819
Echogenicity	1.677	0.843	0.047	5.349	1.025	27.915
Margin	0.560	1.133	0.621	1.751	0.190	16.128
Calcification	0.914	0.354	0.010	2.495	1.246	4.995
Constant	-9.966	2.833	0.000	0.0001		

## Discussion

Lymph node metastasis is one of the factors determining the extent of surgery and use of adjuvant radioactive iodine therapy. This study examined whether the US findings are related to an aggressive pattern of MPTC, such as extrathyroidal extension and LNM. Reported risk factors for LNM include extrathyroidal extension, tumor size, multifocality, and the number of CLNM [11, 13, 24]. All tumors with LCLNM were solid, while most tumors with LCLNM were also solid. Because most of the enrolled tumors were solid in nature, solidity was not a predicting factor for LCLNM, which was shown in the statistical analysis. Hypoechoic or markedly hypoechoic lesions on US were related to LNM and to LCLNM in this study. Other types of calcifications, except microcalcification, are other risk factors for LCLNM, which is in contrast to the finding that microcalcification is suggestive of PTC.

Despite the favorable prognosis of MPTC with survival rates exceeding 90%, locoregional recurrences are not infrequent. LNM at the initial surgery predisposes to recurrence [8, 11, 25], and postoperative adjuvant therapy, including thyroid stimulating hormone suppression therapy and radioactive iodine therapy, reduces the recurrence rate [9]. However, the results reported in the literature are sometimes inconsistent and even conflicting, which is due to the lack of stratification of each risk factor. Although a recent series of studies showed that the presence of nodal metastasis is related to survival [26], the influence of LNM on treatment outcome is limited in the low-risk group, especially in MPTC. CLNM is one of the risk factors for LCLNM and it is unlikely that it affects the distant metastasis and survival rates by itself. Additional factors have been suggested, such as the number of lymph nodes, extranodal spread and node size [27]. While the number of lymph nodes removed at neck dissection is not related to the recurrence rate of PTC [28], LCLNM affects the prognosis in proportion to the primary tumor size [29]. Lateral neck extension is thought to be associated with high rates of recurrence and distant metastasis in MPTC [11, 30, 31]. Radioactive iodine therapy has a limited role in eradicating evident metastasis, where surgical resection is necessary [31]. Therefore, the detection and management of LCLNM is necessary to improve the treatment outcome in MPTC. US findings should be considered when deciding whether to perform surgery or adjuvant therapy because US is the best imaging technique for detecting thyroid nodules. It can also help discriminate benign nodules from malignant ones. Hypoechogenicity, microcalcifications, poorly defined margins, and taller than wide in the transverse plane are suspicious sonographic findings in PTC, and should lead to a US-guided FNA being performed [9, 14].

Although US is an excellent tool for detecting LCLNM in either primary cases or recurrences [32], the clinical implications of the US findings, such as prognosis stratification or predicting aggressive tumor behavior, are not well understood. Previously, we reported that the US findings of PTC are related to extrathyroidal extension and CLNM [18]. In this study, most of the tumors were hypoechoic or markedly hypoechoic (90.9%) and ill-defined (73.2%). The various tumor shapes were distributed evenly. Microcalcification (13.2%) was not detected more frequently than other types of calcification, unlike in other reports. Neither absence of calcification nor microcalcificaion in PTC was a risk factor for LCLNM. It is probably caused by the relatively low detection rate of microcalcification in MPTC in this study. Other types of calcification, including macrocalcification and fine stippled psammomatous calcification, can be risk factors for LCLNM. Underestimating microcalcification and the high proportions of other types of calcification is thought to be associated with these results. However, the potential for LNM varies among these hypoechoic lesions. Therefore, we subdivided hypoechoic lesions into hypoechoic and markedly hypoechoic tumors and this revealed differences in tumor behavior that were significant in the univariate analysis. Since most of LNM in PTC occurs in sequence from the central lymph nodes to the lateral cervical lymph nodes, a hypoechoic lesion with a higher rate of CLNM is also likely to have a higher rate of LCLNM. Large tumor and calcification are risk factors for LNM [33]. However in this study, tumor size in MPTC did not affect the rate of LNM, and the presence of calcification subtypes other than microcalcification was a significant risk factor for LCLNM. Although not significant, most of LCLNMs (n = 13, 92.9%) were found in the tumor with poorly defined margins. Considering that extrathyroidal extension was found in 12 cases, the combined effect of poorly defined margins and extrathyroidal extension may be affect the rate of LCLNM.

Previous studies were based mainly on the diagnostic value of metastatic lymph nodes rather than the significance of each manifestation on US. The sensitivity and specificity for LCLNM are about 90% and 85%, respectively, and vary among different reports [34, 35]. We hypothesized that US findings may predict tumor behavior, and we examined in this study whether the primary tumor characteristics on US are related to LCLNM.

There are some limitations to this study. The diagnostic accuracy of the US findings suggesting malignancy and the direct relationship between the US findings and recurrence rate or survival rate were not evaluated because this study had a cross-sectional design. Despite the limitations, this study identified some pathological features of aggressive primary tumors seen on US that might reflect an increased rate of LCLNM. However, no evident report on tumor biology explains this relationship.

## Conclusions

The primary tumors of MPTC show differences in their US findings. An increased risk of CLNM was reflected mostly in hypoechogenicity or marked hypoechogenicity on US findings for the primary tumor. Despite its low incidence, the risk of LCLNM is significantly related to marked hypoechogenicity and some types of calcification. Markedly hypoechoic lesions might be an indicator of tumor aggressiveness. Further study of tumor biology is needed to explain the correlation between the US findings and pathology.

## Abbreviations

CLNM: central lymph node metastasis
FNA: fine needle aspiration
LCLNM: lateral cervical lymph node metastasis
LNM: lymph node metastasis
MPTC: micropapillary thyroid cancer
PTC: papillary thyroid cancer
US: ultrasonography.
